# *Coxiella*-like bacteria in fowl ticks from Thailand

**DOI:** 10.1186/s13071-018-3259-9

**Published:** 2018-12-27

**Authors:** Wachareeporn Trinachartvanit, Simaporn Maneewong, Warissara Kaenkan, Pawiga Usananan, Visut Baimai, Arunee Ahantarig

**Affiliations:** 1 Biodiversity Research Cluster, Department of Biology, Faculty of Science, Mahidol University, Rama 6 Road, Bangkok, 10400 Thailand; 2 Center of Excellence for Vectors and Vector-Borne Diseases, Faculty of Science, Mahidol University at Salaya, Phutthamonthon 4 Road, Nakhon Pathom, 73170 Thailand

**Keywords:** *Coxiella-*like bacteria, Fowl ticks, Thailand

## Abstract

**Background:**

*Coxiella* bacteria were identified from various tick species across the world. Q fever is a zoonotic disease caused by the bacteria *Coxiella burnetii* that most commonly infects a variety of mammals. Non-mammalian hosts, such as birds, have also been reported to be infected with the pathogenic form of “*Candidatus* Coxiella avium”. This research increases the list of tick species that have been found with *Coxiella-*like bacteria in Thailand.

**Methods:**

A total of 69 ticks were collected from 27 domestic fowl (*Gallus gallus domesticus*), 2 jungle fowl (*Gallus gallus*) and 3 Siamese firebacks (*Lophura diardi*) at 10 locations (provinces) in Thailand. Ticks were identified and PCR was used to amplify *Coxiella* bacteria *16S* rRNA, *groEL* and *rpoB* genes from the extracted tick DNA. MEGA6 was used to construct phylogenetic trees *via* a Maximum Likelihood method.

**Results:**

The phylogenetic analysis based on the *16S* rRNA gene showed that the *Coxiella* sequences detected in this study grouped in the same clade with *Coxiella* sequences from the same tick genus (or species) reported previously. In contrast, *rpoB* gene of the *Coxiella* bacteria detected in this study did not cluster together with the same tick genus reported previously. Instead, they clustered by geographical distribution (Thai cluster and Malaysian cluster). In addition, phylogenetic analysis of the *groEL* gene (the chaperonin family) showed that all *Coxiella* bacteria found in this study were grouped in the same clade (three sister groups).

**Conclusions:**

To our knowledge, we found for the first time *rpoB* genes of *Coxiella-*like bacteria in *Haemaphysalis wellingtoni* ticks forming two distinct clades by phylogenetic analysis. This may be indicative of a horizontal gene transfer event.

## Background

Ticks are ectoparasites of vertebrates transmitting pathogens like protozoa, viruses and bacteria which cause zoonotic diseases in domestic animals and humans. Both hard and soft tick species have been documented to harbour *Coxiella* bacteria. For example, the soft ticks *Ornithodoros capensis* (*s.l.*), *O. rostratus* and the hard ticks *Dermacentor atrosignatus* and *Amblyomma testudinarium* have been found to be infected with *Coxiella* bacteria [1–4].

Q fever (Query fever) is a zoonotic disease caused by *Coxiella burnetii* that most commonly infects a variety of mammals throughout the world. *Coxiella burnetii* affects the reproductive system in animals causing stillbirths and miscarriages. Infection of *C. burnetii* in humans results from inhalation of contaminated aerosols in nature or from direct contact with infected domestic animals products or formites [5, 6]. Individuals infected with *C. burnetii* were detected among rice farmers who raised cattle and chickens in northeastern Thailand. A seroepidemiological survey of *C. burnetii* in cattle and chickens in Thailand was carried out using an indirect fluorescent antibody test. Only one out of 113 serum samples from fowl was seropositive [7]. Moreover, non-mammalian hosts, such as birds, have also been reported to be infected with “*Candidatus* Coxiella avium” [8].

*Coxiella* bacteria were identified from various species of *Haemaphysalis* and *Rhipicephalus sanguineus* (*s.l.*) ticks in Thailand [3, 9]. Nevertheless, in Thailand, infection of *Coxiella* bacteria in fowl ticks has rarely been investigated. Therefore, the aims of this work were to determine the presence of *Coxiella* bacteria in fowl ticks and to study their evolutionary relationships in phylogenetic analyses based on partial *16S*, *rpoB* (RNA polymerase beta-subunit), and *groEL* (the chaperonin family) gene sequences.

## Methods

### Tick samples and identification

A total of 69 ticks were collected from 27 domestic fowl (*Gallus gallus domesticus*), 2 jungle fowl (*Gallus gallus*) and 3 Siamese firebacks (*Lophura diardi*) at 10 locations (provinces) in Thailand during 2014–2016: (i) Chaiyaphum; (ii) Chumphon; (iii) Krabi; (iv) Pattani; (v) Rayong; (vi) Satun; (vii) Songkhla; (viii) Surat Thani; (ix) Trang; and (x) Yala (Table 1). The ticks were removed by using forceps, stored in 70% alcohol and preserved at -20 °C awaiting further identification and molecular assays. Ticks were classified by developmental stage and sex and identified based on morphology using standard identification keys [10, 11]. Ticks positive for *Coxiella* bacteria were also molecularly identified using a primer set consisting of 16S + 1 and 16S - 1 to detect tick *16S* mitochondrial DNA (*16S* mDNA) [12].

**Table 1:** Tick samples from domestic fowl (*Gallus gallus domesticus*), jungle fowl (*Gallus gallus*) and Siamese fireback (*Lophura diardi*)

Location no. / Province	No. of hosts	No. of ticks tested			Tick species	No. of ticks positive for *Coxiella*
		M	F	N		
1. Chaiyaphum	Siamese fireback (*n* = 3)	0	0	3	*A. testudinarium*	1N
		0	1	0	*H. wellingtoni*	–
		0	0	5	*H. obesa*	5N
2. Chumphon	Domestic fowl (*n* = 2)	1	2	0	*H. wellingtoni*	1F
3. Krabi	Domestic fowl (*n* = 3)	3	2	0	*H. wellingtoni*	1M, 1F
		0	0	3	*H. bispinosa*	1N
4. Rayong	Domestic fowl (*n* = 1)	0	1	0	*H. wellingtoni*	1F
5. Satun	Domestic fowl (*n* = 5)	5	4	0	*H. wellingtoni*	1F
		0	0	3	*H. wellingtoni*	–
6. Trang	Domestic fowl (*n* = 2)	0	2	0	*H. wellingtoni*	2F
7. Pattani	Domestic fowl (*n* = 2); jungle fowl (*n* = 1)	0	2	0	*H. wellingtoni*	–
		5	6	0	*R. microplus*	–
		0	0	3	*H. wellingtoni*	–
8. Songkhla	Domestic fowl (*n* = 9)	7	4	0	*H. wellingtoni*	–
9. Surat Thani	Domestic fowl (*n* = 2)	2	0	0	*H. wellingtoni*	–
		0	0	1	*H. wellingtoni*	–
10. Yala	Domestic fowl (*n* = 1); jungle fowl (*n* = 1)	0	1	0	*H. wellingtoni*	–
		1	2	0	*R. microplus*	–
Total	32	24	27	18		14

Ticks were obtained from 10 locations in Thailand. The number of ticks tested and positive results for *Coxiella* bacteria are shown. *Abbreviations*: M, male; F, female; N, nymph; *A*., *Amblyomma*; *H*., *Haemaphysalis*; *R*., *Rhipicephalus*

### DNA extraction from tick samples and PCR

Ticks were washed with 70% ethanol and 10% sodium hypochlorite and rinsed three times with sterile distilled water. Then, ticks were immediately homogenized using the TissueLyser system (QiagenGmbH, Hilden , Germany). One 3-mm tungsten carbide bead (Qiagen GmbH, Germany) was added to each tube (collection microtubes; Qiagen GmbH, Germany) and ticks (individual for adult, individual for *Amblyomma* nymph and a pool of 5 for nymph of *Haemaphysalis*) were homogenized for 4 min at 30 Hz. After a short centrifugation step (5 s at 3220× *g*), the supernatants were collected in separate collection microtubes and DNA extracted using Qiagen’s DNeasy Blood and Tissues Kit (Qiagen GmbH, Germany) following the manufacturer’s instructions. Genes and primers used to amplify *Coxiella* DNA were used as in a previously reported protocol [13].

### Purification and sequencing of PCR products

After PCR amplification and gel electrophoresis, DNA bands corresponding to positive amplification results were excised. Purified DNA samples (using Purification kit from Roche, Basel, Switzerland) were sent to the Ramathibodi Research Department (Ramathibodi Hospital, Bangkok, Thailand) for DNA sequencing. The results were analysed and compared with other DNA sequences from GenBank in the National Center for Biotechnology Information database (NCBI: https://blast.ncbi.nlm.nih.gov/Blast.cgi?PAGE_TYPE=BlastSearch).

### Phylogenetic analyses

DNA sequences were edited and aligned with MEGA6 using ClustalW multiple sequence alignment algorithm. DNA sequences from this study, along with selected reference strains from GenBank, were used to construct a phylogenetic tree *via* Maximum Likelihood (Kimura 2-parameter model) and determining the confidence value for each branch of the phylogenetic tree with bootstrap analysis by using 1000 pseudoreplicates of the original alignment.

## Results and Discussion

A total of 69 ticks collected from domestic and jungle fowl and Siamese firebacks belong to 3 genera: *Haemaphysalis*; *Amblyomma*; and *Rhipicephalus* (Table 1). The 51 adult ticks included 14 *R. microplus*, 37 *H. wellingtoni* and the remaining 18 ticks were nymphs of *H. wellingtoni* (*n* = 7), *H. obesa* (*n* = 5), *H. bispinosa* (*n* = 3) and *A. testudinarium* (*n* = 3). The *16S* mDNA sequences of ticks were submitted to the GenBank database under the accession numbers MG865746 (*H. wellingtoni*), MG874025, MG910463 and MG874022 (*H. obesa*, *H. bispinosa*, and *A. testudinarium*, respectively). A total of 14 out of 69 ticks tested were positive for *Coxiella* bacteria, as defined by the amplification of *16S* rRNA sequences. Positive results were found in *H. wellingtoni* from Chumphon, Krabi, Rayong, Satun and Trang, *H. obesa* from Chaiyaphum, *H. bispinosa* from Krabi and *A. testudinarium* from Chaiyaphum (Table 1).

**Fig. 1:**
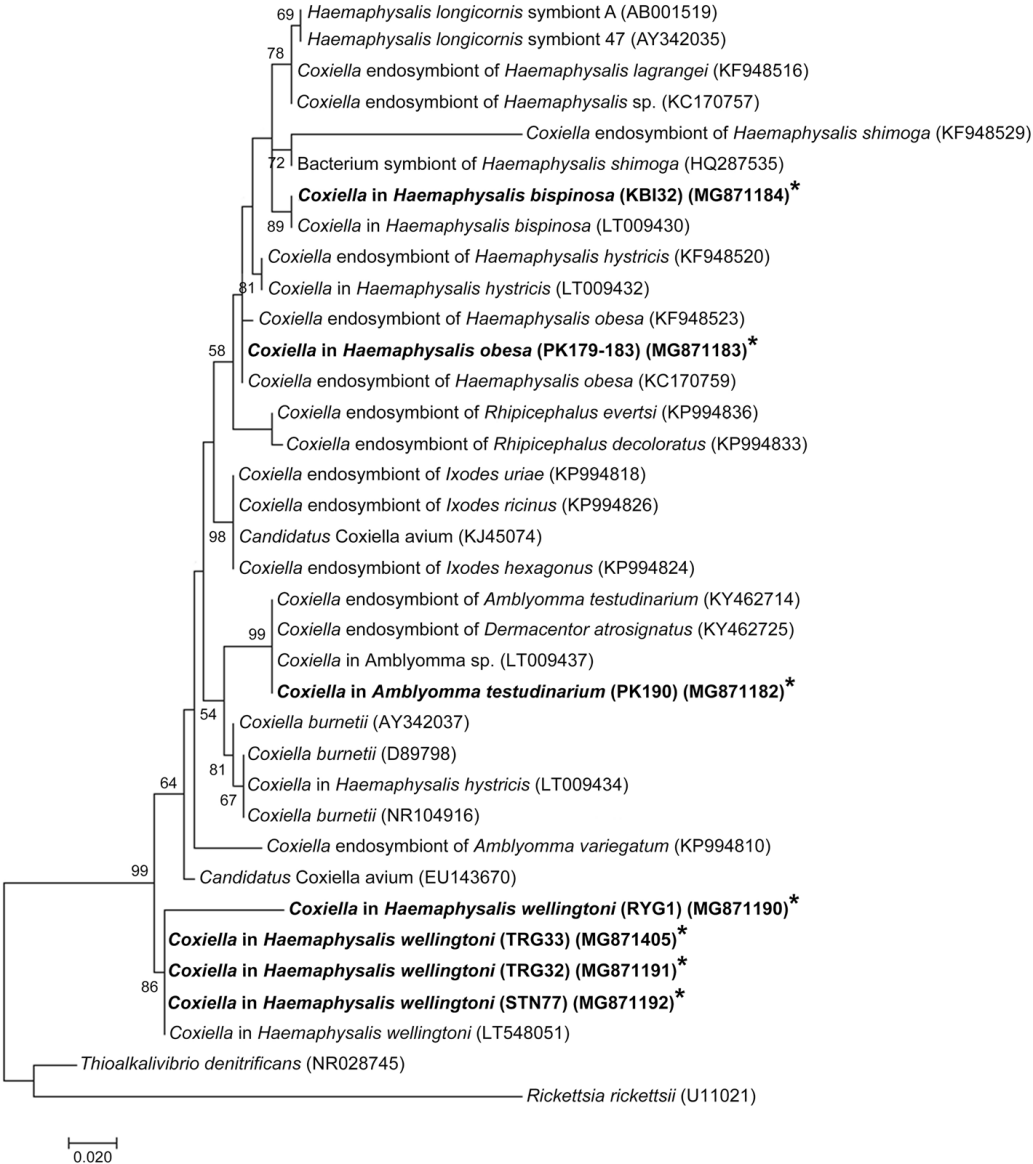
Phylogenetic tree for *Coxiella*-like bacteria *16S* rRNA gene sequences constructed with the Maximum Likelihood method using MEGA6 software. Bootstrap analysis was performed with 1000 pseudoreplicates. *Rickettsia rickettsii* was used as the outgroup. *Coxiella-*like bacteria isolates from this study are indicated in bold and with asterisks

*Coxiella* bacteria-positive samples were sequenced, and a phylogenetic tree was constructed based on their analysis (Fig. 1). *Coxiella* DNA sequences were submitted to the GenBank database, including *H. wellingtoni* from Trang (TRG32 and TRG33), *H. obesa* and *A. testudinarium* from Chaiyaphum (PK179-183 and PK190), *H. wellingtoni* from Rayong (RYG1), *H. wellingtoni* from Satun (STN77) and *H. bispinosa* from Krabi (KBI32) (see Fig. 1 for accession numbers).

The phylogenetic analysis, based on the *16S* rRNA gene, showed that *Coxiella* bacteria from *Haemaphysalis* ticks of domestic fowl and Siamese firebacks were grouped with *Coxiella* bacteria of the same corresponding tick species previously reported (Fig. 1). In addition, the *16S* rRNA sequence of *A. testudinarium* of our study was in the same group with those reported by Nooroong et al. [3] and Khoo et al. [14] (Fig. 1).

**Fig. 2:**
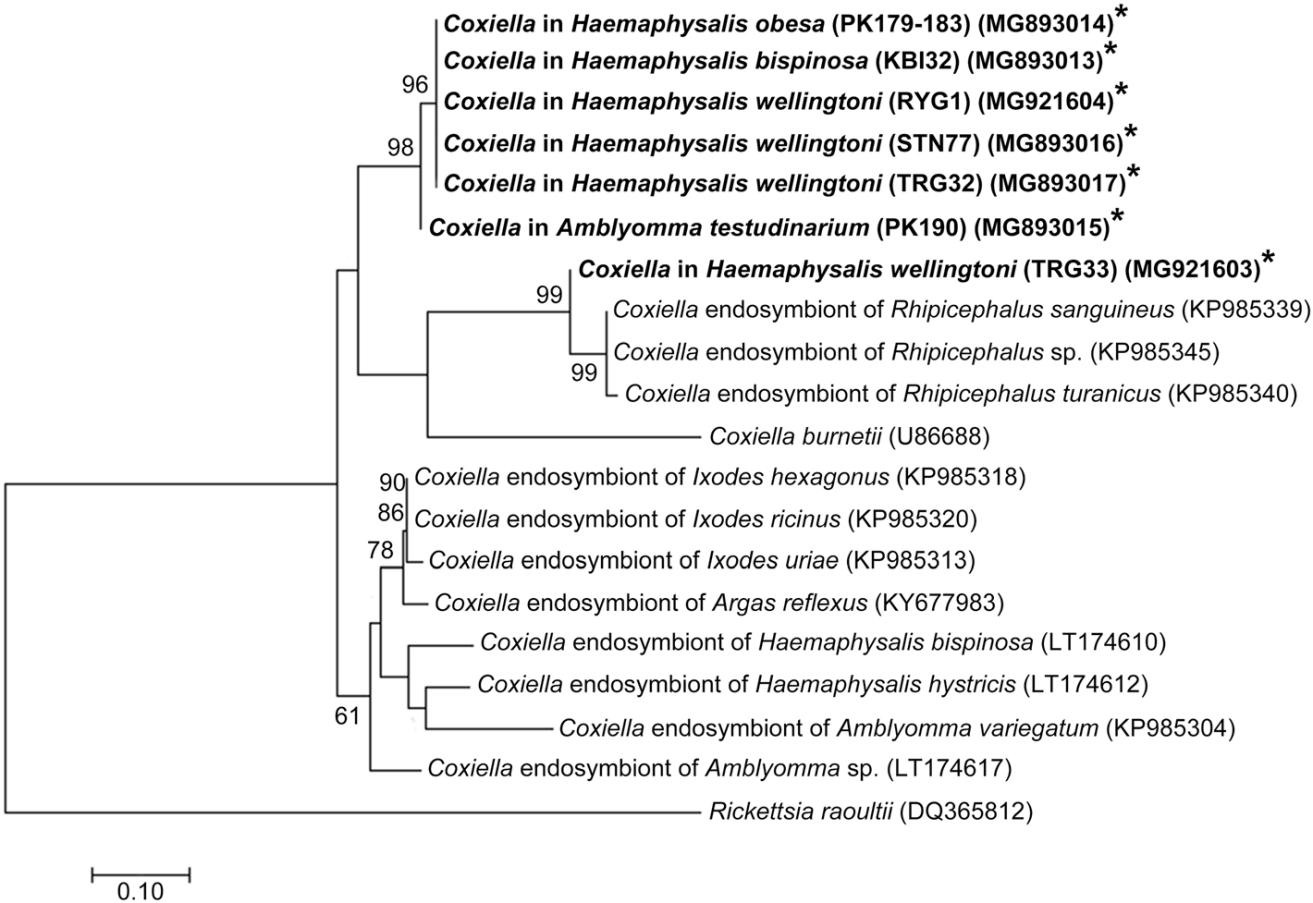
Phylogenetic tree for *Coxiella*-like bacteria *rpoB* gene sequences constructed with the Maximum Likelihood method using MEGA6 software. Bootstrap analysis was performed with 1000 pseudoreplicates. *Rickettsia raoultii* was used as the outgroup. *Coxiella-*like bacteria isolates from this study are indicated in bold and with asterisks

**Fig. 3:**
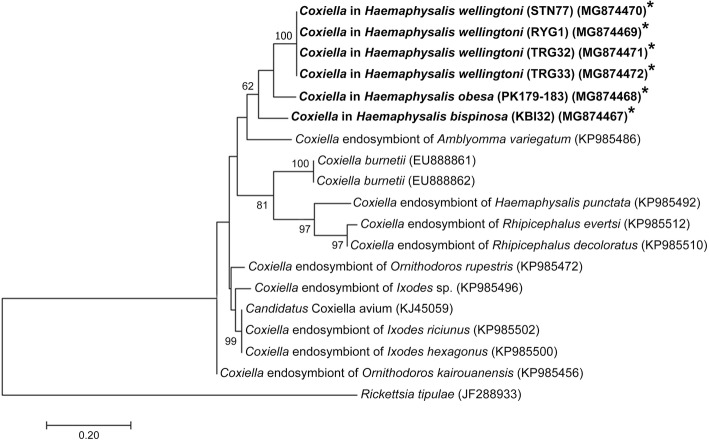
Phylogenetic tree for *Coxiella*-like bacteria *groEL* gene sequences constructed with the Maximum Likelihood method using MEGA6 software. Bootstrap analysis was performed with 1000 pseudoreplicates. *Rickettsia tipulae* was used as the outgroup. *Coxiella-*like bacteria isolates from this study are indicated in bold and with asterisks

Phylogenetic analyses of *rpoB* and *groEL* genes of *Coxiella*-like bacteria were also performed. The results are shown in Figs. 2 and 3. Most of *Coxiella rpoB* sequences from this study were in the same group and exhibited 88–89% identity with *Coxiella*-like endosymbiont of *Argas reflexus* (isolate Areflex2, GenBank: KY677983) and *Coxiella* endosymbiont of ticks of the genus *Ixodes* (GenBank: KP985313, KP985318 and KP985320) (Fig. 2). However, *rpoB* gene sequences of *Coxiella-*like bacteria in *H. wellingtoni* from Trang (TRG33) clustered in a different clade and was closely related to *Coxiella* endosymbiont of *Rhipicephalus* sp. isolate (Tchien14; GenBank: KP985345; 96% identity) (Fig. 2). The *rpoB* gene sequences of *Coxiella* bacteria detected in this study did not cluster together with those previously reported in the same tick species by Khoo et el. [14] (*H. bispinosa* and *Amblyomma* spp.). Instead they seemed to be clustering by their geographical distribution forming a Thai cluster (the present study) and a Malaysian cluster (data by Khoo et al. [14]). *Coxiella groEL* gene sequences detected in ticks from domestic fowl and Siamese firebacks from this study were clustered in the same clade (three sister groups) and exhibited about 89% DNA sequence identity with “*Candidatus* Coxiella avium” from seabird ticks (GenBank: KJ459059) (Fig. 3).

*Coxiella* bacteria have been reported in several tick species, such as *Rhipicephalus sanguineus* (*s.l.*), *Amblyomma americanum*, *Ixodes uriae* and the soft tick *O. rostratus* [1, 2, 15, 16]. In addition, *Coxiella*-like bacteria were also detected in *Haemaphysalis* ticks, such as *H. lagrangei*, *H. obesa*, *H. shimoga* and *H. hystricis* [3]. In the present study, the rate of *Coxiella-*like bacteria in ticks collected from fowl was rather high because about 20% of ticks of the 4 species (*H. wellingtoni*, *H. bispinosa*, *H. obesa* and *A. testudinarium*) were positive for *Coxiella*-like bacteria. Thus, our results seem to agree with those of Arthan et al. [4] who demonstrated that the prevalence was not dependent on tick species.

Analyses of *rpoB* sequences revealed that most of *Coxiella-*like bacteria exhibited 88–89% identity with *Coxiella*-like endosymbiont from *Argas reflexus* (isolate Areflex2). However, *rpoB* gene of *Coxiella* bacteria in *H. wellingtoni* from Trang (TRG33) was clustered in the different group in the phylogenetic analysis and related to *Coxiella-*like bacteria of *Rhipicephalus* sp. isolate Tchien14 rpoB gene, partial cds (96% identity). This result may simply be indicative of a horizontal gene transfer event. Since only a small number of sequences is reported here, and only one with these characteristics is shown, it remains to be determined what is the real impact of this observation. The interesting point is that *Coxiella rpoB* sequences from different *H. wellingtoni* ticks belong to a different clade (even from the same tick species). The roles of these *Coxiella-*like bacteria in ticks and their fowl hosts are still unclear and needs further investigation.

## Conclusions

To our knowledge, we found for the first time that *Coxiella rpoB* gene sequences from different *H. wellingtoni* ticks belong to two different clades and that *rpoB* sequence of the *Coxiella* bacteria detected in this study did not cluster together with those previously reported in the same tick species.
